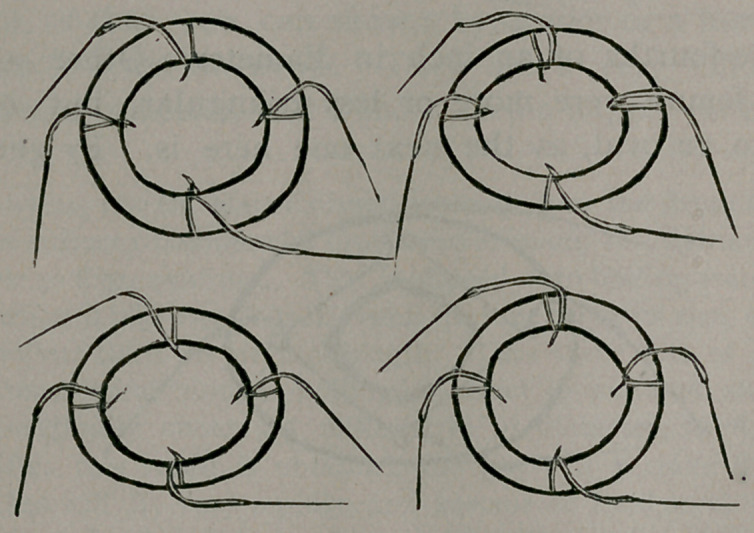# Some Observations upon the Experimental Use of the Aided Intestinal Suture

**Published:** 1891-11

**Authors:** Geo. H. Lee


DANIEL'S

### TEXAS MEDICAL JOURNAL.

A Representative Organ of the Medical Profession, and an Exponent of Rational Medicine; devoted to the Organization, Advancement and Elevation of the Profession in Texas.

PUBLISHED MONTHLY.—SUBSCRIPTION $2.00 A YEAR.

Vol. VII. AUSTIN, NOVEMBER, 1891. No. 5.

### Original Articles.

CONTRIBUTED EXCLUSIVELY TO THIS JOURNAL.

*The Articles in this Department are accepted and published with the understanding that we are not responsible for, nor do we indorse the views and opinions of the writers by so doing.*

---

For Daniel's Texas Medical Journal.

### SOME OBSERVATIONS UPON THE EXPERIMENTAL USE OF THE AIDED INTESTINAL SUTURE.

BY GEO. H. LEE, M. D.

Read before the Galveston County Medical Club, October 12th, 1891.

IN THE immense mass of medical literature daily being produced, much of which is of great value, there is nothing of more interest or of more importance than the recent work of Prof. Nicholas Senn upon "Intestinal Surgery."

So important were the results of his experiments in this domain, and so easily apparent was the value of the ideas he advanced, and the facts that he submitted regarding the aided intestinal suture, that the profession received them with enthusiasm, and many operators of ability have been working in this field. The new facts observed have been eagerly sought and rapidly disseminated until it is almost impossible at first thought to believe the subject of aided intestinal suture has been before the profession only for about four years.

Various substances have been suggested as aids to substitute the original decalcified bone plate of Prof. Senn, all of which are fully and carefully described by the very able article of Dr. Randolph Matas, in the New Orleans *Medical and Surgical Journal* for August and September, 1890. No material however has been found which so fully meets the necessary demands under all circumstances, at every different portion of the canal, as the decalcified bone plate.

It is not my purpose this evening to enter upon a general discussion of the aided intestinal suture, or of the various materials which have been used as aids—subjects and matter which are familiar to the profession.

During the past spring and summer I have been making some operations upon dogs, with the idea simply of familiarizing myself with the details of intestinal suture by the aided method of Senn, and especially with the difficulties encountered in such surgery. It is to a brief presentation of certain points observed during these operations which I would ask your attention. As an introduction I would like for sake of review simply to enumerate the indications which Prof. Senn proposes to meet with his decalcified bone plate. They can be very well grouped under three heads, viz :

1st. Time saving: To produce a method which can be used quickly, and which will not lengthen an operation as under old methods.

2nd. Security: To prevent perforation or giving way of the walls of the bowels and leakage.

3rd. Preservation of the lumen of the intestine and the avoidance of intestinal obstruction.

The question of time in all abdominal operations is universally conceded of primary importance. The immensely prolonged operations with the unaided Czerny-Lembert suture was responsible for a mortality which is appalling, and which under the aided methods has happily been much lowered. The decalcified plates ready prepared are slipped into the canal, the walls of the intestines transfixed, the threads tied, and the intestine easily and quickly surrounded by a continuous Lembert suture, which is not difficult of execution because the walls are held accurately in position by the rings. I was able to make in this way a circular enterorraphy in a cadaver in the dead house of John Sealy Hospital in slightly less than ten minutes, although the third time I had attempted the operation. The greatest impediment which I

have found in using the aids are the 4 to 6-8 inch long silk threads tied on each ring. With all the usual tendency of silk to tangle these numerous threads will, in spite of the greatest care, very frequently become snarled and knotted and tangled at the most inopportune moment, and once so it is almost impossible to bring order out of chaos. Time will be saved by laying that ring aside and using another when the operation is again liable to the same accident. In my last experiment, made some six days ago with the aid of Prof. Morris and the staff of Sealy Hospital, I sought to avoid this accident by the use of silk worm gut, and was much pleased with the result. Silk worm gut is non-absorbable like silk, is capable of being rendered aseptic, and will not tangle. It ties easily and nicely in a very pliable knot, and is simply suggested as a very desirable substitute for the silk.

Each suture of silk worm gut can be armed with a needle as used with the silk suture by Senn and others; but I prepared to use only one needle—a patent self-threading needle—into which I slipped each suture as I passed them through the bowels. A very small especially-made Skene's needle might be even more convenient and used more quickly.

Security against perforation, or giving away and leakage, is most efficiently produced by the aided suture. The rings within the intestinal canal, with the sutures transfixing the walls of the intestine, serve to hold the serous surfaces in apposition after the manner of bone clamps. For this reason as a result from my observations, I am not disposed to concede that the only purposes of the ring are subserved after the Lembert sutures are introduced. They have then served only one of their purposes, *i. e.*, that of facilitating the introduction of this suture. They should keep their form for several days, to still act as clamps holding the serous coats in apposition, and preserving the lumen of the intestine to prevent obstruction, as occurred by too speedy collapse of the ring in one of my cases in which the Matas drum-snare-ring was used. In fulfilling this office all rings of cat-gut, or cat-gut plates, or drum-snare are faulty as giving way too quickly under contact of the intestinal juices. The ends of the ring should be flat to hold larger portions of the serous surfaces in contact. For this reason rings made of round material as the drum-snare of Matas, or catgut wrapped after Abbe's method, or the rings of drainage tube suggested by Brokaw are not so efficient as the decalcified bone plate of Senn, the catgut mats of

Davis, and the rawhide plates of Robinson. The security of this junction can be easily illustrated on the cadaver where it is not difficult to make a circular enterorraphy that will hold water perfectly.

The scarifications of those portions of the serosa that are brought in contact with the needle or sharp instrument is considered of great importance by the different operators.

The importance of preserving the lumen of the intestine, has been very generally noted and dwelt upon. Especially in intestinal anastomosis, where the wall of the intestine is simply slit open, it is necessary for the rings to keep their shape until union is firm, and until the edges of the incision into the bowel have begun to granulate. At the same time, to introduce a material into the canal which would not undergo absorption, or would collapse too slowly, would simply augment the danger to be avoided.

My experiments and my operations upon the cadaver, have been most largely with the Matas ring of drum-snare, a number of which, made after his* directions, are here for your observation. He speaks of preparing them in a few moments. It is true their preparation does not require very long, but do not deceive yourselves with the idea that you can make them as you need them, or that you can show a bystander or a horse how to make them in a few moments. They are a very efficient ring, in the absence of better, and I shall keep a supply on hand, just

---

*The following are Dr. Matas' directions for making the drum snare rings: He uses the ordinary commercial drum snare, made for tightening drums, as preferable to finer qualities. This is dipped into boiling water for a few moments, when it will uncoil itself, swell to three times its size, and become shortened to one-third its original length. Thus three feet of drum snare will, after two or three minutes boiling, contract to one foot, and the cord, which was originally about 3½ millimetres in diameter, swell to 6 millimetres. After this result is obtained, the gut will remain permanently shortened, but will dry very rapidly, and become as hard as wood. It may be then immersed indefinitely in water, and it will simply soften to the consistency of a solid rubber band, but will never show a disposition to become distorted by kinking or coiling. With a material thus prepared, it is easy to make rings of any desired size by simply cutting and shaping them; furthermore, they are made to retain their shape by simply inserting the free ends in a small piece of rubber tubing, which acting as a clasp, is sufficient to keep the ring in shape; in order to secure the tubing permanently, it will be safer to tie the ring to the tubing with silk thread. The ring is then ready to be mounted with the fundamental or perforating sutures." (N. O. Med. & Surg. Journal, August, 1890.)

as catgut is kept. I have also here two catgut rings made after Abbe's directions. These rings have, as you are aware, been proven efficient by successful operations upon man. I do not see that they are more efficient than the Matas ring, or Brokaw's ring of segments of drainage tube. These two rings consumed several hours in their preparation, after I had been informed by Aloe & Co., of St. Louis, that they could not be obtained ready made. I have ordered twice from the druggist in Milwaukee, who is said to prepare them under Dr. Senn's direction,—the decalcified bone plates,—but, am sorry to say, have never heard from my letters.* I now present you some rings which may have been, probably have been, used by a number of operators in this line, and yet are not mentioned in any article or book which I have been able to command. This one is made from a transverse section of the femur of a year old veal. It is round, about three-fourths of an inch in diameter. Other sections of the same femur were more or less triangular, but could be trimmed to an oval, as the next one here is. By getting the femur at different ages, up to the adult animal, almost any size ring can be made. I have adopted the direction of Pro. Senn in their preparation, except that I made transverse sections, trimming out the medullary canal for the central opening, instead of taking sheets from the hard wall of the femur and cutting the

*These rings were in the express office at the time, and through neglect of the express company had not been delivered.

central opening out of the sheet. My idea of preparation is then as follows: I take the femur of an adult beef, on down to a veal —according to the size of ring desired—clean it of periosteum, and with a sharp saw make tranverse sections of the bone about one-eighth of an inch thick. These sections are immersed in diluted muriatic acid until thoroughly decalcified. Then they are placed in a very weak solution of liquor potassa, to neutralize the acid. They are trimmed then with a sharp knife, into regular circle, or oval, and are ready to receive the sutures. I submit that these rings possess all the desirable features of the Senn decalcified bone plates, as described by him. They are not so broad on the ends, and for this reason do not present such large spaces for the coaptation of the serous surfaces, but the central opening is very much larger than in the Senn ring, and the rings are sufficiently firm and hard to furnish the desired resistance and support. They are very easy of preparation, and in assorted sizes, from four to six sutures of silk worm gut fastened to each, should be kept on hand in dilute alcohol, by every surgeon who essays, or who may be compelled in any emergency to do intestinal surgery. Here are some rings thus prepared, that have been in alcohol for the past four weeks, and are in excellent condition.

The knots in the silk worm gut should be carefully arranged, so that they are entirely on the inside of the ring, and the short end of the suture cut close. The sutures should all emerge from the same side of the ring, and in introducing the ring into the lumen of the bowel, I regard it as important to let this side, from which all the sutures come, enter the bowel first, so that the sutures will transfix the walls of the intestine from behind the rings. at it were, thus catching a firmer hold, and bringing the peritoneum together more nicely.

I am confident a little experience with the use of these rings will convince any one that a simple needle in a handle, as a very fine needle made after Skene's, or a patent self-threading needle, is just as convenient, and can be used just as quickly, as a needle on each string, where they are continually getting in the way. The Lembert suture is easily and quickly made with an ordinary sewing needle and soft silk.

During the course of my modest experiments, I made several tests of the use of hydrogen gas in the diagnosis of intestinal perforation, and was much pleased with the result. To my mind, the profession is under great obligation to the distinguished Prof. Senn for his careful, painstaking and eminently successful labors, and I regard his experiments in this field as the most important pioneer work of the present generation, so justly proud of its remarkable progress in surgery.

## Figures and Tables

**Figure f1:**
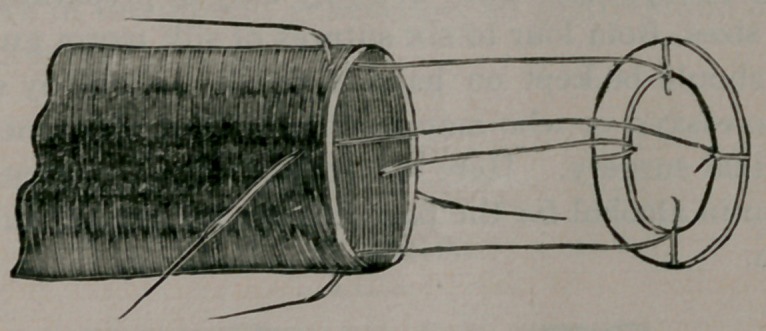


**Figure f2:**
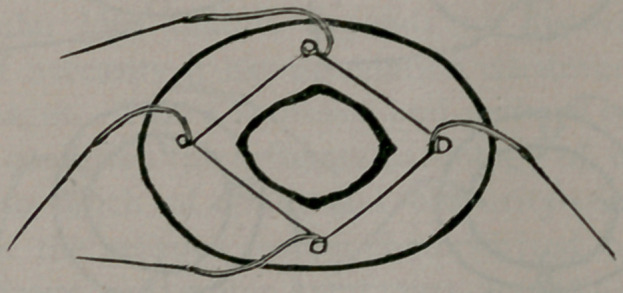


**Figure f3:**